# Prediction, prevention, and precision treatment of immune checkpoint inhibitor neurological toxicity using autoantibodies, cytokines, and microbiota

**DOI:** 10.3389/fimmu.2025.1548897

**Published:** 2025-03-13

**Authors:** Alberto Vogrig, Marta Dentoni, Irene Florean, Giulia Cellante, Rossana Domenis, Donatella Iacono, Giacomo Pelizzari, Simone Rossi, Valentina Damato, Martina Fabris, Mariarosaria Valente

**Affiliations:** ^1^ Department of Medicine (DMED), University of Udine, Udine, Italy; ^2^ Clinical Neurology, Department of Head-Neck and Neuroscience, Azienda Sanitaria Universitaria Friuli Centrale (ASUFC), Udine, Italy; ^3^ Institute of Clinical Pathology, Department of Laboratory Medicine, Azienda Sanitaria Universitaria Friuli Centrale (ASUFC), Udine, Italy; ^4^ Department of Oncology, Azienda Sanitaria Universitaria Friuli Centrale (ASUFC), Udine, Italy; ^5^ IRCCS - Istituto delle Scienze Neurologiche di Bologna, Bologna, Italy; ^6^ Department of Neurosciences, Drugs and Child Health, University of Florence, Firenze, Italy

**Keywords:** neurologic adverse events, neurological complications, Guillain-Barré syndrome, limbic encephalitis, autoimmune encephalitis, paraneoplastic neurological syndromes, myositis, myasthenia gravis

## Abstract

Cancer immunotherapy with immune checkpoint inhibitors (ICIs) has revolutionized oncology, significantly improving survival across multiple cancer types. ICIs, such as anti-PD-1 (e.g. nivolumab, pembrolizumab), anti-PD-L1 (e.g. atezolizumab, avelumab), and anti-CTLA-4 (e.g. ipilimumab), enhance T cell-mediated anti-tumor responses but can also trigger immune-related adverse events (irAEs). Neurological irAEs (n-irAEs), affecting 1-3% of patients, predominantly involve the peripheral nervous system; less commonly, n-irAEs can present as central nervous system disorders. Although irAEs suggest a possible correlation with treatment efficacy, their mechanisms remain unclear, with hypotheses ranging from antigen mimicry to cytokine dysregulation and microbiome alterations. Identifying patients at risk for n-irAEs and predicting their outcome through biomarkers would be highly desirable. For example, patients with high-risk onconeural antibodies (such as anti-Hu or Ma2), and elevated neurofilament light chain (NfL) levels often respond poorly to irAE treatment. However, interpreting neuronal antibody tests in the diagnosis of n-irAEs requires caution: positive results must align with the clinical context, as some cancer patients (e.g., SCLC) may have asymptomatic low antibody levels, and false positive results are common without tissue-based confirmation. Also, the use of biomarkers (e.g. IL-6) may lead to more targeted treatments of irAEs, minimizing adverse effects without compromising the anti-tumor efficacy of ICIs. This review provides a comprehensive overview of the latest findings on n-irAEs associated with ICIs, with a focus on their prediction, prevention, as well as precision treatment using autoantibodies, cytokines, and microbiota. The most interesting data concern neuronal antibodies, which we explore in their pathogenic roles and as biomarkers of neurotoxicity. Most of the available data on cytokines, both regarding their role as diagnostic and prognostic biomarkers and their role in supporting therapeutic decisions for toxicities, refer to non-neurological toxicities. However, in our review, we mention the potential role of CXCL10 and CXCL13 as biomarkers of n-irAEs and describe the current evidence, as well as the need for further studies, on the use of cytokines in guiding selection of second-line therapies for n-irAEs. Finally, no specific microbiome-related microbial signature has been proven to be linked to n-irAEs specifically, leading to the need of more future research on the topic.

## Introduction

Cancer immunotherapy using immune checkpoint inhibitors (ICIs) has revolutionized the field of oncology by significantly extending the survival of patients across various cancer types (1). To understand how these drugs work and their potential adverse events, it is essential to first discuss the regulation of the immune system, particularly T cells, under normal conditions. T cells play a critical role in protecting the body from pathogens and neoplasia, but their activity must be carefully regulated to prevent autoimmunity (1). T cell activation requires three key steps (three-signal model): (i) antigen recognition, the presentation of an antigen via major histocompatibility complex molecules to the T cell receptor, (ii) co-stimulation between co-stimulatory molecules on APC and CD28 receptor on the T cell, (iii) cytokines, with regulatory and differentiation functions. Recently, a fourth step has been proposed to highlight the importance of the metabolic/nutritional status of T cells, particularly in the context of the tumor microenvironment (2).

Conversely, the immune response can also be inhibited by evolutionarily conserved negative regulators like cytotoxic T-lymphocyte antigen-4 (CTLA-4) or programmed death receptor-1 (PD-1), after binding with their ligands, namely CD80 and PD-1 ligand (PD-L1), respectively (3, 4). Tumor cells may exploit these mechanisms by overexpressing checkpoint ligands like PD-L1, thereby suppressing the immune response. Blocking these interactions with monoclonal antibodies (Abs) targeting PD-1 (e.g., nivolumab, pembrolizumab), PD-L1 (e.g., atezolizumab, avelumab), CTLA-4 (e.g., ipilimumab), and more recently, Lymphocyte Activation Gene-3 (LAG-3) (e.g., relatlimab) has transformed cancer treatment (3, 4).

While ICIs can induce powerful anti-tumor responses, they can also cause immune-related adverse events (irAEs), which may affect any organ system, including the nervous system in 1-3% of patients (3). Patients with severe irAEs need hospitalization, discontinuation of therapy with ICIs, and treatment with immunosuppressants. In some cases, irAEs can be fatal (5), and fatality rate is high for neurological irAEs (n-irAEs) (6). More than 50% of the patients with n-irAEs who survive the acute phase develop a chronic condition (7).

Although there is some evidence suggesting a link between irAEs occurrence and the effectiveness of ICIs (5), the underlying mechanisms of these toxicities still need to be clarified. Several hypotheses have been proposed to explain these adverse events, including antigen mimicry between cancer and self-antigens (8), development of neoantigens and subsequent breakdown of immune tolerance (9), cytokine dysregulation (10), and microbiome alterations (11). Given the rapid expansion of ICIs use, it is crucial both for neurologists and oncologists to become familiar with the diagnosis and management of neurological irAEs.

Neurological toxicities involve the peripheral nervous system three times more frequently than the central nervous system (CNS) (6). The most common disorders, in order of frequency, are myositis, peripheral neuropathies, myasthenic syndromes, encephalitis, and cranial neuropathies (6). Other CNS manifestations include demyelinating disorders (6) and cerebellar irAEs (12). Many of these disorders are associated with neuronal Abs in ICI-naïve patients, but their role in the context of cancer immunotherapy has not yet been systematically explored.

Currently, we are unable to accurately predict either the onset of toxicity or the potential toxicity phenotype. However, preferential associations between the type of toxicity, cancer, and ICIs have been reported in the literature. The available data are frequently derived from clinical studies/case series with a limited number of patients, due to the rarity of neurological toxicities; studies are often retrospective and heterogeneous in terms of the definition of the clinical phenotype and analysis of different patient subgroups. Despite these limitations, similar evidence has emerged across different patient cohorts. For example, Farina et al. (13) analyzed the frequency of clinical toxicity phenotypes in relation to the type of ICI used, and observed that in patients treated with anti-PD-L1 therapy, myositis and limbic encephalitis phenotypes predominated, while in patients treated with anti-CTLA-4 +/- an anti-PD-L1, polyradiculoneuropathy and meningitis phenotypes were more prevalent. These correlations were also observed in another study (6), which highlighted that in patients with myasthenic syndromes the use of anti–PD-1/PD-L1 was more prevalent, in the cohort of patients with meningitis the use of anti–CTLA-4 predominated, while in patients who developed cranial neuropathies exposure to anti-PD-1/PD-L1 and anti-CTLA-4 was more frequent compared to the entire examined sample. A higher frequency of neuromuscular junction dysfunction in patients treated with anti-PD-1/PD-L1 compared to those treated with anti-CTLA-4 was also observed in a large cohort of patients (14); in the same cohort, Guillain-Barré syndrome and non-infectious meningitis were more frequent in the group of patients treated with combined anti-PD-L1 and CTLA-4 therapy compared to monotherapy. Regarding cancer type and non-irAE associations, lung cancer was significantly more frequent in patients with CNS toxicities and paraneoplastic-like syndromes (e.g., limbic encephalitis), while melanoma was more common in those with peripheral neuropathies or meningitis (13, 15).

Identifying patients at increased risk of autoimmunity through biomarkers would be highly desirable, allowing for preemptive intervention to reduce the risk of irAEs without compromising the anti-tumor efficacy of ICIs (16). Serum biomarkers are particularly valuable because they can be easily obtained with minimal discomfort for the patients, and serum analysis can be conducted rapidly and cost-effectively (5). Moreover, the use of effective biomarkers could facilitate more targeted treatments, aiming to resolve irAEs without a negative impact on cancer prognosis.

This review provides a comprehensive overview of the latest findings on n-irAEs associated with ICIs, with a focus on their prediction, prevention, as well as precision treatment using auto-Abs, cytokines, and microbiota.

## How ICIs may induce the production of auto-Abs: the role of B cells

Checkpoint molecules play a crucial role not only in regulating T cell tolerance but also in the function of B cells (16). Upon encountering an antigen, B cells can differentiate into plasma cells, which are specialized in secreting large quantities of Abs. This process highlights why certain neuronal Abs may be detected following treatment with ICIs. However, the clinical significance of these Abs can vary, ranging from a mere epiphenomenon to actively contributing to disease development (3).

Recently, the correlation between T cell receptor (TCR)/B cell receptor (BCR) and irAEs has been investigated. Che et al. (17) analyzed TCR and BCR repertoires in a cohort of patients with basal cell carcinoma treated with anti-PD-1 (18) and observed a significant variability both across patients and between tumor samples taken before and after PD-1 inhibitor treatment in the same patient. From a predictive point of view, they identified an increase in unique BCR clonotype counts in the surviving patient group after anti-PD-1 administration. Lozano et al., instead, observed an increased pretreatment diversity of the TCR repertoire in a cohort of metastatic melanoma patients treated with anti-PD-1 monotherapy or anti-PD-1 and anti-CTLA-4 combination ICIs, who developed severe irAEs (19). Further studies are needed to validate and explore the predictive role of BCR and TCR in the development of neurological toxicities. The observation of a link between ICIs and B cells is also sustained by substantial evidence that supports the role of CTLA-4 and PD-1 in the modulation of B cells (16).

Patients with heterozygous CTLA-4 germline mutations develop B cell alterations and have an increased risk of autoimmunity (20), including cases with neurological involvement (21). The effect of ICIs on B cells appears to be more pronounced when combinations of different drugs are adopted. Das et al. analyzed changes in circulating B cells before and after the first cycle of therapy in patients with advanced melanoma receiving ICIs (16). Patients treated with combined checkpoint blockage (anti-CTLA-4 and anti-PD-1) experienced a significant decrease in the total number of circulating B cells, along with greater increase in plasmablasts compared to patients in the monotherapy-treated cohorts (16). Corroborating these findings, combination therapy led to a greater increase in plasma C-X-C motif chemokine ligand 13 (CXCL13) levels, a recently described marker of germinal center activation (22), which is crucial for the development of a robust and specific immune response. Also, combination therapy led to distinct early changes in B cells, including an increase in the distinct B cell subset known as CD21low B cells. CD21low B cells are potent antigen presenting cells because of the high baseline surface expression of co-stimulatory markers, contributing to the activation and cytokine release from CD4 T cells (23). It is therefore not surprising that CD21low B cells are expanded in many autoimmune diseases, such as systemic lupus erythematosus and rheumatoid arthritis (23), therefore potentially representing an early marker of irAEs. Conversely, changes in circulating T, NK, and myeloid cell numbers after therapy do not correlate with a risk of irAEs (16).

## Prediction of n-irAEs

### Neuronal autoantibodies

Since autoreactive B cells can be activated following ICI treatment, auto-Abs are likely to play a significant role in irAEs, including n-irAEs. These auto-Abs could be valuable in predicting risk of such toxicities (if they are present before treatment), as well as in early diagnosis and prognosis. Regarding the latter, it should be considered that some auto-Abs are pathogenic (e.g. neuronal surface or synaptic Abs), directly contributing to the disease, while others serve as biomarkers of a destructive T cell-mediated immune response (e.g. onconeural or so called “high risk” Abs), with obvious implications for patient outcomes.

(a) CNS irAEs. Among irAEs affecting the CNS, three main phenotypes were identified: limbic encephalitis (LE), meningoencephalitis, and cerebellitis (15). LE triggered by ICIs show clinical (seizures, psychiatric symptoms, working memory deficits), and neuroradiological (T2-weighted fluid-attenuated inversion recovery magnetic resonance imaging (MRI) alterations restricted to the medial temporal lobes) evidence of a focal encephalitis affecting the limbic system (15, 24). This syndrome is otherwise indistinguishable from ICI-naïve LE, which can be paraneoplastic (e.g. in the context of anti-Hu or anti-Ma2 autoimmunity) or non-paraneoplastic (e.g. as in anti-LGI1 encephalitis). Indeed, the same paraneoplastic “high-risk” Abs (e.g., anti-Hu, anti-Ma2) can be found in LE triggered by cancer immunotherapy, suggesting a common immunopathological background between this type of irAEs and naturally occurring paraneoplastic neurological syndromes (PNS). Moreover, in both scenarios, the presence of onconeural Abs generally implicates a poor prognosis. Conversely, the occurrence of neuronal surface or synaptic Abs associated with the most common form of autoimmune encephalitis (i.e. NMDAR Abs), which generally responds favorably to treatment, is very rare after ICIs (6).

ICI-related meningoencephalitis, a non-focal syndrome with altered mental status and pleocytosis, has distinctive features in terms of (i) immunological associations (patients are either Ab-negative, harbor Abs against unknown antigenic specificities, or develop Abs against glial fibrillary acidic protein - GFAP); (ii) oncological associations (most of the patients do not harbor neuroendocrine cancers); (iii) greater likelihood of treatment response; (iv) diagnostic findings (e.g., patients are less likely to show MRI abnormalities and often present more pronounced cerebrospinal fluid (CSF) inflammatory findings as compared to focal encephalitis); (v) more frequent association with concurrent non-neurological irAEs.

Interestingly, ICI-related cerebellar toxicity, a rarer disorder, is also different from its paraneoplastic counterpart (i.e. paraneoplastic cerebellar ataxia, PCA) due to different demographic and clinical aspects (male patients with lung cancer in the former, women with gynecological/breast cancers in the latter), as well as immunological findings (heterogeneous and less-characterized Abs such as tripartite motif-containing protein 9 (TRIM9) or neurofilament light chain (NfL) Abs in cerebellar irAE versus well-characterized high-risk Abs, especially anti-Yo, in the PCA group), and outcome (better outcome in cerebellar irAE as compared to PCA) (12).

When approaching a patient with suspected n-irAEs, there are several caveats in the interpretation of neuronal Ab testing: (i) a positive Ab result should always be interpreted in combination with the clinical picture, since patients with certain cancers (e.g. small-cell lung cancer, SCLC) may asymptomatically harbor low neuronal Ab titers (e.g. anti-Hu) in 16–22% of cases (25); (ii) false positive test results are common if commercial kits are used alone, without the required confirmation using tissue-based assays (26); (iii) Hu and Ma2-Abs may be detected retrospectively in pre-ICI serum samples, indicating that these Abs can be present prior to the development of immune-related neurological symptoms (suggesting a “double hit hypothesis”) (25, 27). Taken together, these findings indicate that a subset of patients with SCLC may develop a subclinical, low-titer anti-neuronal immune response, which could be exacerbated to a clinically apparent level after treatment with ICIs (25, 28). However, since only a minority of these patients will develop the anti-Hu or anti-Ma2 neurological syndrome, this should not be considered a criterion for excluding them *a priori* from cancer immunotherapy. Other relevant factors are likely involved, such as specific Human Leukocyte Antigen (HLA) haplotypes. For example, a study found that 3/5 (60%) patients with atezolizumab-induced meningoencephalitis harbored the rare HLA-B*27:05 allele (29).

(b) Peripheral nervous system irAEs. In peripheral nervous system disorders triggered by ICIs the role of auto-Abs might be more relevant in the setting of myasthenic syndromes, where 32/56 cases (57%) were positive for anti-Acetylcholine Receptor (anti-AChR) in a previous study, while anti-Muscle-Specific Kinase (anti-MuSK) or anti-Voltage-Gated Calcium Channel (anti-VGCC, the biomarker of Lamber Eaton myasthenic syndrome) were detected only exceptionally (6). Despite such a significant prevalence (which is, however, significantly lower than in ICI-naïve myasthenia gravis, MG), it is still unclear whether anti-AChR Abs are pathogenic in the setting of immune-related neuromuscular junction dysfunction. In two patients with anti-AChR positive immune-related MG, the auto-Abs did not harbor effector functions (i.e., complement fixation and blocking properties) (30). Additionally, patients with myositis may show low-titer AChR Abs despite lacking clinical or neurophysiological evidence of neuromuscular junction dysfunction (6, 31), therefore physician should refrain from giving a diagnosis of ICI-related MG based on the positive AChR Abs result alone. Apart from AChR Abs, the presence of myositis-specific and myositis-associated Abs is otherwise uncommon in muscular disorders triggered by ICIs (6). On the other hand, anti-striated muscle Abs (e.g., titin Abs) can be found in patients with thymoma-related MG (32). Similarly, anti-titin Abs were detected in cases triggered by ICIs in which myasthenia and myositis overlap, suggesting a possible pathophysiological similarity between ICI-induced and thymoma-associated autoimmunity. Notably, a subgroup of patients can develop the severe “triple M” syndrome, when also myocarditis adds to the clinical picture (6). Intriguingly, 4/5 patients with thymoma who had pre-existing AChR Abs, were shown to develop myositis after anti-PD-L1 treatment (33). In agreement with this finding, Suzuki and colleagues demonstrated that the majority of cancer patients with MG induced by treatment with nivolumab exhibited preexisting AChR Abs (34). From a broader perspective, it has been proposed that neuromuscular auto-Abs, including anti-titin, anti-skeletal muscle, anti-cardiac muscle, anti-Lipoprotein Receptor-Related Protein 4 (LRP4), anti-Ryanodine Receptor (anti-RyR), and anti-AChR, could be used as biomarkers for the diagnosis and potential prediction of ICI-induced neuromuscular diseases, such as myositis and severe MG (35).

Some authors recommend testing for AChR Abs before initiating ICI treatment in patients with thymoma (31). In our experience, this testing is particularly useful when thymoma is a secondary cancer and ICIs are being used to treat another malignancy, such as lung cancer. This is especially important given that the use of ICIs in thymoma itself is relatively rare.

Finally, auto-Abs are detected in a minority (10/42, 24%) of patients with ICI-related Guillain-Barré-like syndrome, the most frequent being anti-gangliosides (in particular GM1 and GM2) (6).

### Systemic autoantibodies

As irAEs tend to be more prevalent in patients with autoimmune diseases or pre-existing auto-Abs, it has been suggested that Ab-mediated autoreactivity could be a predictive marker and/or a causal factor (36). In a recent study, a customized array of 162 antigens was used to analyze the level of auto-Abs in cancer patients at baseline and during ICI therapy. Compared to healthy controls, patients already had a greater number of IgG and IgM reactivities prior to ICI administration, and those who manifested irAEs demonstrated pre-ICI IgG reactivity to a higher number of autoantigens than patients who did not develop irAEs (37). Additionally, microarray analysis of 120 autoantigens commonly associated with autoimmune disease demonstrated that melanoma patients who experienced specific irAE had fewer expressed auto-Abs at baseline than those that did not have irAE, but a greater fold change in Ab concentration from baseline to 6 weeks linked to specific irAE development. However, no auto-Abs were identified as being predictive of specific events (38). Further studies are needed to assess whether these considerations also apply to n-irAEs.

### NfL, S100B, and GFAP

GFAP and NfL are well-validated biomarkers of astroglial and neuronal injury, respectively. GFAP is an intermediate filament of astrocytes and its up regulation in the blood or CSF is a specific marker of astrocyte activation and/or injury. For example, in case of traumatic brain injury, GFAP levels reflect the clinical severity (39). NfL has emerged as an excellent biomarker of neuronal injury in many neurologic conditions, including multiple sclerosis, dementia, stroke, and amyotrophic lateral sclerosis and is being applied to monitor disease including assessment of treatment efficacy (40). In a single-center retrospective cohort study, the analysis of brain damage markers S100 calcium-binding protein B (S100B) and NfL concentration in blood showed high sensitivity and specificity for early detection and monitoring of CNS irAEs in cancer patients treated with combined CTLA-4 and PD-1 blockage (41). High levels of NfL were found in serum and CSF of patients with severe immune-related encephalitis associated with nivolumab/ipilimumab, and serum NfL levels reflected the clinical course better than MRI findings. These findings suggest that NfL levels might be utilized as an additional monitoring parameter during ICI treatment (42). In a recent study, serum NfL levels in ICI-encephalitis were found to be comparable to Herpes simplex virus (HSV) encephalitis, and effectively distinguished patients with definite ICI-encephalitis from cancer-matched controls, as well as treatment responders from non-responders (24). Collectively, these findings suggest that serum NfL levels could be valuable for identifying patients who may need to discontinue ICI treatment and undergo further diagnostic procedures (e.g., lumbar puncture, brain MRI, EEG, and neural Ab testing) to confirm the diagnosis and initiate immunosuppression (24). This is particularly important because a subset of patients (12-20%) with ICI-related encephalopathy may not exhibit neuronal Abs or inflammatory changes in CSF/brain MRI but still respond to immunosuppressive therapy (24, 43). However, it is crucial to approach this diagnosis with caution, as several alternative diagnoses, such as neoplastic leptomeningeal disease, infectious conditions, and metabolic disorders, must be thoroughly excluded before attributing symptoms to neurological toxicity from ICI (44, 45). Unlike NfL, serum S100B levels (also a melanoma biomarker) only differentiated definite ICI-encephalitis from cancer-matched controls after excluding patients with melanoma. Finally, serum GFAP levels did not distinguish between any of the groups (24).

### Cytokines

Cytokines are pleiotropic immune-regulators with extensive biological activities which play key roles in controlling cell development, growth, survival, and differentiation, through autocrine or paracrine pathways (46). In the tumor microenvironment, all types of cytokines are involved in intercellular communications including interleukins (ILs), interferons (IFNs), the tumor necrosis factor (TNF) superfamily, chemokines, and growth factors (47). Numerous studies have been aimed at identifying a possible correlation between systemic cytokines levels and ICI efficacy (48) or onset of irAEs (49). Pre-therapeutic assessment of inflammation and cytokine profiles may predict response and survival in patients with non-small-cell lung cancer treated with single ICI agent. Specifically, patients with elevated neutrophil-to-lymphocyte ratio, signs of systemic inflammation and increased IL-6 and IL-8 levels showed significantly lower response to ICI treatment and reduced progression free survival. Conversely, elevated levels of interferon-γ (IFN-γ) defined a subgroup of patients who significantly benefits from ICI treatment (50). In a broader context, an important observation was that lower baseline levels of specific cytokines at onset (low level of systemic inflammation) and their excessive upregulation after ICI treatment (suggestive of ICI-related T cell activation) is closely related to the occurrence of irAEs. In a recent study in which a large panel of cytokines and chemokines were assessed in sera of patients receiving ICIs, a significant upregulation of CXCL9, 10, 11 and 13 was closely related to the occurrence of immune-mediated toxicities including pneumonitis, endocrinopathies, dermatitis, arthritis and encephalitis (10). Furthermore, an elevated level of certain cytokines was found prior to ICI treatment in melanoma patients developing severe irAEs, suggesting that ICI treatment amplified an unrecognized subclinical inflammatory status (51). In detail, the CYTOX score, which includes proinflammatory cytokines such as IL1a, IL2, and IFNα2, was proposed as a new tool for predicting severe adverse reactions that may help in the early management of toxicity in patients with melanoma treated with combination anti-CTLA-4 and anti-PD-1 (51). To date, few studies investigated the possible correlation between cytokine levels and n-irAEs triggered by ICIs, probably also due to their lower incidence as compared to gastrointestinal, hepatic, or lung toxicities. Elevated levels of interleukin 17 (IL17A), but no other cytokines, have been associated with immune-related neuroendocrine toxicity, characterized by insulin-dependent diabetes, hypophysitis and a myasthenic-like syndrome in a patient with sarcomatoid mesothelioma treated with dual immune checkpoint blockade (anti-PD-1 and anti-TIM3 Abs) (52). Finally, an immune signature indicative of enhanced chemotaxis and inflammation was also identified by multiplex cytokine assay in ICI-treated cancer patients with high-grade n-irAE, identifying CXCL10 as a promising prognostic biomarker, showing the strongest increase in n-irAEs patients compared to controls, the highest levels detected during acute illness and a significant rise in the setting of multiorgan immunotoxicity (53). The increase of CXCL13 (a key player involved in T cell-B cell interaction required for B cell activation) has also been hypothesized as a potential biomarker, both in central and peripheral neurological toxicities (54).

### Microbiota

Growing evidence reveals that gut microbiota plays a role in shaping host immunity, influencing both the innate and the adaptive immune system. Pattern recognition receptors (PRRs) expressed on epithelial and innate immune cells in the intestine recognize pathogen-associated molecular patterns (PAMPs), triggering an intracellular cascade of events culminating in the transcription of inflammatory mediators (55). Microorganisms are therefore capable of influencing specific intracellular pathways. On the other hand, in the gut-associated lymphoid tissue (GALT), PRRs stimulation also induces naïve B and T cells priming and subsequent further differentiation (55).

Not only does gut microbiota determine host immunity, but also it plays a role in tumor response and autoimmune complications related to ICI therapy (56). However, considering the complexity of microbiome-immune system interaction, drawing well-established conclusions on how gut microbial composition influences ICI clinical response or the development of irAE is extremely difficult: several variables come into play influencing gut microbiome, including diet, medication use, geography, and ethnicity (56).

(a) Protective role against irAEs. Several studies have linked certain bacterial species or phyla to increased or decreased susceptibility to irAEs. Bacteria belonging to the Bacteroidetes phylum have been demonstrated to be protective against the development of ICI-related colitis in metastatic melanoma patients treated with anti-CTLA-4 (57, 58), though possibly correlating with a poorer oncological outcome (57). Similarly, Bacteroides and Parabacteroides (Bacteroides phylum), together with Phascolarctobacterium (Firmicutes phylum) were shown to negatively correlate with immune-mediated diarrhea in a group of advanced lung cancer patients treated with anti-PD-1 (59). The protective role of Bacteroidetes seems to be confirmed by the identification of decreased abundance of Bacteroides species in inflamed intestinal regions of solid cancer patients developing immune-related colitis after receiving anti-PD-L1 treatment (60). Baseline relative abundance of Bacteroides vulgatus was also associated with a lower risk of irAEs in advanced melanoma patients treated with ICI combination therapy; however, the same authors demonstrated that Bacteroides dorei was linked to an increased risk of irAEs, implying that individual species belonging to the same genus may have a differential impact on irAEs (61). Gut enrichment in Bifidobacterium and Desulfovibrio genera also appears to be protective in lung cancer patients undergoing single anti-PD-1/PD-L1 immunotherapy (62). Bifidobacterium, together with Enterobacter spp. and an unclassified genus of Erysipelotrochaceae were also associated with reduced toxicity to combination ICI (anti-PD-1 plus anti-CTLA-4 blockade) (63); the same work showed a possible role of Dialister sp. in patients with reduced irAEs to nivolumab.

(b) Increased susceptibility to irAEs. While certain bacterial species seem to play a protective role against irAEs, some others have been linked to increased susceptibility to irAEs of variable severity. Enrichment in Bacteroides intestinalis and Intestinibacter bartlettii in baseline gut microbiome samples of patients exposed to combined ICI blockade correlated with development of ≥grade 3 irAEs (64). In patients with advanced lung cancer receiving anti-PD-1/PD-L1 therapy, one study demonstrated that Akkermansia species was associated with a lower severity of irAEs, while Agathobacter correlated with more severe irAEs, as well as better clinical outcomes (65). In another work, Veillonella (Proteobacteria phylum) prevailed in patients experiencing diarrhea (59). Lachnospiraceae spp. and Streptococcaceae spp. were both associated with irAEs in a cohort of melanoma patients treated with anti-PD-1; interestingly, the two species were associated with opposite effects on ICI clinical response (favorable and unfavorable, respectively) (66). Higher relative abundance of Streptococcus was also demonstrated on fecal samples during combined (anti-CTLA-4 plus anti-PD-1) ICI treatment, when patients developed severe (i.e., ≥grade 3) irAE; enrichment in pro-inflammatory genera Escherichia-Shigella was also demonstrated, both in baseline fecal samples and during treatment (67). However, such differences were not confirmed in patients undergoing single-agent anti-PD-L1 treatment. A third study pointed out the relevance of Streptococcus in irAEs, stating that patients treated with anti-PD-1 (in combination with either chemotherapy or antiangiogenic agents) and developing severe irAEs showed enrichment in Streptococcus, Faecalibacterium, and Stenotrophomonas, as compared with patients without or with mild irAEs (grades 0–2). In the latter group, Faecalibacterium and unidentified Lachnospiraceae prevailed (68). Interestingly, the same authors tried and develop a classification model based on five microbial biomarkers (Actinomyces graevenitzii, Dorea formicigenerans, Bacteroides ovatus, Bacteroides finegoldii, Lachnospiraceae bacterium) which managed to distinguish patients without irAEs from those with severe irAEs with a good predictive power (68).

(c) Predictive role in immunotherapy response. Other than the ability to predict irAEs following ICI exposure, gut microbiota signature may predict oncological response to immunotherapy (57, 60, 66, 69–77). It is likely that primary resistance to ICIs could be related to dysbiosis itself, as confirmed by the fact that fecal microbiota transplant from ICI responders to non-responders was able to overcome resistance in a subset of patients (78, 79).

Though evidence is preliminary, the role of microbiota is doomed to expand in the near future, possibly contributing to a higher quality management of the oncological patient undergoing immunotherapy. To our knowledge, no microbial signature has so far been linked to n-irAEs specifically, which therefore represents an interesting area of future research.

## Implications for the prevention and precision treatment of n-irAEs

The selection of immunomodulatory or immunosuppressive therapy for patients who develop n-irAE is currently driven by the nature and severity of the toxicity (80). The identification of prognostic factors for n-irAEs and predictive factors for response to immunosuppressive therapy is certainly a desirable goal to achieve. Investigating the potential role of specific biomarkers in guiding treatment choices for neurological toxicity could be highly valuable, especially given the widespread availability of many of these biomarkers:

### Cytokines

According to the ESMO guidelines (80), if severe toxicity and refractoriness to first-line neurologic immunotherapy (corticosteroids, intravenous immunoglobulin, plasmapheresis) occur, then a second-line therapy, such as rituximab, cyclophosphamide, tocilizumab or infliximab, may be considered. Drug selection is individualized and based on patient characteristics including comorbidities, presence of other irAEs, and potential impact on cancer prognosis. It is reasonable to assume that tocilizumab may be efficacious in patients with CNS toxicity and elevated IL-6 levels in serum and/or CSF (81). However, in a recent study, seven patients with focal encephalitis, including 4/5 with elevated CSF interleukin-6 (IL-6) levels, were treated with tocilizumab, but only one (14%) showed significant improvement (24), possibly due to the inability to cross the blood-brain barrier. It is important to note that there is currently no clear evidence supporting the use of CSF IL-6 levels as a marker for predicting treatment response to these drugs. The use of IL-6 blockade has shown more promising results in ICI-related myopathy, where transcriptomic analyses of muscle biopsy samples revealed that overexpression of IL-6 pathway genes is a key feature distinguishing immune-related myopathy from healthy controls and various forms of idiopathic inflammatory myopathy (82). Tocilizumab may also be considered to prevent irAEs in high-risk patients. In a recent study, two patients with pre-existing autoimmune disorders, including dermatomyositis and giant cell arteritis, were treated with tocilizumab prophylaxis to prevent disease flare after ICI (83). Of particular interest, the first patient, who had active underlying paraneoplastic dermatomyositis and stage IV melanoma, received one dose of anti-PD-1 and subsequently developed a severe flare with musculoskeletal involvement and elevated C-reactive protein levels, requiring immunosuppressive treatment. Due to disease progression, the patient was re-challenged with low-dose anti-PD-1 following prior administration of tocilizumab, and the dermatomyositis did not flare (83). To understand the risk of relapse in this setting, another study reported that dermatomyositis was exacerbated after ICI in 3 out of 4 patients with pre-existing PNS who did not receive preventive tocilizumab (84). In addition, tocilizumab also proved its efficacy in treating ICI-associated arthritis and in the prevention of relapses after rechallenge (85). It would be interesting to assess a similar use of tocilizumab in n-irAEs.

Infliximab (anti-TNFα) is currently used for diverse irAEs, including gastrointestinal and rheumatological toxicities, in addition to corticosteroids (80, 86, 87), with the potential benefit of shortening the duration of corticosteroid therapy. In isolated reports, infliximab was used to treat CNS n-irAEs, without strong evidence of efficacy (15, 44). However, both infliximab and tocilizumab are potential options as second-line immunosuppressive therapies due to their selectivity of action and their probable absence of impact on cancer prognosis (86, 88, 89). Further studies will be required to confirm these hypotheses.

### Neuronal antibodies

In clinical practice, intracellular or surface neuronal Abs are not routinely assessed in patients being considered for ICI therapy. However, multiple studies have shown that the presence of high-risk Abs, along with their associated clinical PNS phenotype (90), is linked to a higher risk of n-irAEs and poor neurological outcomes (27, 44). This suggests a need for increased vigilance in the early diagnosis of n-irAEs and a more aggressive treatment approach, with early initiation of second-line therapies, especially when such high-risk Abs are detected (44). Specific neuromuscular Abs could potentially serve as predictive factors for development of neuromuscular irAEs (i.e. myositis and MG). However, these biomarkers could also play a role in therapeutic decision-making (35). Since T cell-mediated mechanisms prevail in patients with ICI-induced neuromuscular disease and striated muscle auto-Abs, T cell targeted therapies could be chosen. Instead, the presence of anti-AchR Abs in patients with ICI-induced neuromuscular disease might be pathogenic: in this subgroup, B cell depletion or plasma exchange treatment may be more indicated. Although the precise molecular mechanism remains unclear, it appears that ICIs may unleash the activation of autoreactive CD4+ T-cells, which, by assisting B-cell activation, lead to autoantibody production, such as the anti-AchR and anti-skeletal muscle Abs (32, 91).

### T-cell biomarkers

Considering the similarities between PNS triggered by ICI treatment and classical PNS, it is logical to assume that the same immunopathological mechanisms are shared by these conditions. For example, tumors (SCLC) of patients with anti-Hu PNS show overexpression of T-cell- and IFNγ-related signatures (92). Specifically, the analysis of transcriptomic profiles revealed that genes upregulated in the anti-Hu group were ZBP, which is involved in the production of type I interferon, and XCR1, which is involved in the connection between innate and adaptive immune response by presenting extracellular antigens to CD8+ T cells (92). STAT1 signaling, which boosts the cytotoxic activity of CD8+ T cells in response to type I interferons, and CCL2, which regulates the activation and recruitment of phagocytes in the CNS, have been shown to play a role in other T-cell mediated neuroimmunological disorders (93). Similarly, IFNγ-induced activation of JAK–STAT signaling seems to be central to the development of n-irAEs. The JAK–STAT pathway is, therefore, a potential therapeutic target for irAEs (31) but, at the same time, it is also relevant for ICI-mediated anti-tumor T cell effector function. Two recently published clinical trials support such hypotheses: combined JAK inhibition and immune-checkpoint blockade in Hodgkin lymphoma patients who relapsed or were refractory following ICI treatment, was able to rescue and enhance the efficacy of ICI (94). Similar results were demonstrated in SCLC, where JAK inhibition was administered after immune-checkpoint blockade, improving antitumor response in mice (95). However, contrary to previous findings related to ruxolitinib and itacitinib, retrospective data suggest an increased risk of cancer in patients with rheumatoid arthritis treated with the JAK inhibitor tofacitinib (96). Finally, it is important to emphasize that if a diagnosis of PNS can be established, initiating therapy with ICI would lead to an exacerbation of clinical symptoms, a development of severe n-irAEs and a negative outcome (35, 97).

### Microbiota

As previously mentioned, microbiota composition plays a role in the prediction of irAEs, as it has been linked to increased or decreased susceptibility to irAEs. Regulating the abundance of beneficial bacteria by modulating microbiota composition, may therefore reduce the risk of immune-related toxicity. Several interventions have been explored, including fecal microbiota transplantation (FMT), probiotics and prebiotics administration, and dietary interventions; however, while several data confirm the potential of such interventions (most especially FMT) to improve immunotherapy efficacy and overcome ICI resistance (55, 98), little is known on how modulation of gut microbiota may prevent irAEs and guide precision treatment. To date, few data exist on the use of FMT in ICI-related colitis (99, 100), while to our knowledge gut microbiota role has not been explored in n-irAEs.

This represents an area of interest for future research, as demonstrated by preliminary evidence in other neuroimmunological disorders. Short-chain fatty acids supplementation in multiple sclerosis patients has been proven effective in reducing annual relapse rate, disability progression, and brain atrophy (101, 102). In the mouse model, FMT from healthy donors was able to improve MG and experimental autoimmune encephalitis symptoms (103, 104).

## Conclusions

Cancer immunotherapy, particularly with ICIs, has become a vital tool in oncology, offering better tolerance compared to traditional chemotherapy. However, the rise of irAEs, especially neurological toxicities (rare, but associated with a high mortality rate), underscores the need for better predictive and preventive strategies. **Table 1** provides a summary of the current knowledge and limitations of the role of biomarkers and microbiota in predicting, diagnosing, monitoring and defining the prognosis of n-irAEs. The identification of serum biomarkers could enable early detection of patients at risk for autoimmunity, allowing for tailored interventions that minimize irAEs without compromising the anti-cancer effects of ICIs. As the field evolves, precision treatments based on biomarkers, auto-Abs, cytokines, and microbiota offer a promising approach to enhance patient outcomes while preserving cancer prognosis.

**Table 1:** Role of biomarkers and microbiota in predicting, diagnosing, monitoring and defining the prognosis of neurological immune-related adverse events after treatment with immune checkpoint inhibitors.

	Biomarker	Comment	Advantages	Limitations
Prediction	**Pre-existent neuronal Abs**	Autoreactive B cells can be activated following ICI treatment, auto-Abs are likely to play a significant role in irAEs, including n-irAEs	High-risk, paraneoplastic antibodies could predict n-irAE development	Neuronal Ab testing must be interpreted in combination with the clinical picture; false positive results with commercial kits (without confirmatory tissue-based assays); SCLC patients may develop low-titer anti-neuronal immune response (e.g. Hu Abs), but only a minority will develop a neurological syndrome
	HLA-B*27:05 allele	/	Harboring such allele may predict the development of specific n-irAEs (i.e. encephalitis)	Under investigation, limited evidence
	**Systemic autoantibodies**	irAEs tend to be more prevalent in patients with autoimmune diseases	Ab-mediated autoreactivity could be a predictive marker and/or causal factor	No systemic auto-Abs are predictive of specific irAEs; lack of data on n-irAEs
	**Cytokines**	Consider type, baseline levels and upregulation after ICI treatment	Significant increase over low baseline levels of CXCL9, 10, 11 and 13 was related to encephalitis development	Limited evidence in n-irAEs
	**Microbiota**	Gut microbiota plays a role in shaping host immunity, influencing both the innate and the adaptive immune system	Certain bacterial species or phyla may predict increased or decreased susceptibility to irAEs	Lack of evidence in n-irAEs; the complexity of microbiome-immune system-external variables interaction makes it difficult to pinpoint its role in irAEs development
Early diagnosis	**NfL, S100B and GFAP**	They are biomarkers of astroglial and neuronal injury	Blood and/or CSF concentrations may serve for early detection of CNS irAEs	Lack of specificity, lack of well-established thresholds for significance
Monitoring	**NfL, S100B and GFAP**	/	Blood and/or CSF concentrations may serve for monitoring of CNS irAEs	/
Prognosis	**High-risk Abs**	/	They generally implicate a poor prognosis in the appropriate clinical context (e.g. Hu Abs)	/
Precision treatment	**Cytokines**	May help in choosing the most appropriate second-line immunotherapy	Elevated IL-6 levels in serum and/or CSF may support tocilizumab use	Limited evidence
	**Neuronal antibodies**	High-risk Abs along with their associated clinical PNS phenotype are linked to a higher risk of n-irAEs and poor outcome	Their presence indicates the need of increased vigilance and early initiation of second-line immunotherapies	/
		T cell-mediated mechanisms prevail in patients with ICI-induced neuromuscular disease and striated muscle auto-Abs, while anti-AchR Abs may be pathogenetic	Prefer B-cell targeted therapies when anti-AchR Abs are present, otherwise T-cell directed therapies should be chosen	/

The limitations of current research include lack of data on microbiota and its possible role in the prediction, prevention and treatment of n-irAEs specifically. Evidence is still preliminary for cytokines and biomarkers of neuronal/astroglial injury, and further exploration of these fields is needed.

In the future, routine screening for neural Abs commonly associated with paraneoplastic neurological syndromes should be considered ahead of starting ICI treatment. In addition, future research should focus on pathological data, including tissue biopsies and autopsies of fatality cases, leading to better understanding of the mechanisms of immune-mediated toxicity and improved management of n-irAEs. Knowledge of pathogenetic mechanisms of n-irAEs could also be helpful in deepening our current understanding of “spontaneous” neuroimmunological disorders. In addition, recommendations on safety of rechallenge after n-irAEs are lacking; risk assessment and prevention of relapses during rechallenge represents an area of great interest for upcoming research.

## References

[1] Waldman AD Fritz JM Lenardo MJ. A guide to cancer immunotherapy: from T cell basic science to clinical practice. Nat Rev Immunol. (2020) 20:651–68. doi: 10.1038/s41577-020-0306-5 PMC7238960 32433532

[2] Giles JR Globig A-M Kaech SM Wherry EJ. CD8+ T cells in the cancer-immunity cycle. Immunity. (2023) 56:2231–53. doi: 10.1016/j.immuni.2023.09.005 PMC11237652 37820583

[3] Vogrig A Muñiz-Castrillo S Desestret V Joubert B Honnorat J. Pathophysiology of paraneoplastic and autoimmune encephalitis: genes, infections, and checkpoint inhibitors. Ther Adv Neurol Disord. (2020) 13:175628642093279. doi: 10.1177/1756286420932797 PMC7318829 32636932

[4] Weinmann SC Pisetsky DS. Mechanisms of immune-related adverse events during the treatment of cancer with immune checkpoint inhibitors. Rheumatology. (2019) 58:vii59–67. doi: 10.1093/rheumatology/kez308 PMC6900913 31816080

[5] Nuñez NG Berner F Friebel E Unger S Wyss N Martinez Gomez J. Immune signatures predict development of autoimmune toxicity in patients with cancer treated with immune checkpoint inhibitors. Med. (2023) 4:113–129.e7. doi: 10.1016/j.medj.2022.12.007 36693381

[6] Marini A Bernardini A Gigli GL Valente M Muñiz-Castrillo S Honnorat J. Neurologic adverse events of immune checkpoint inhibitors. Neurology. (2021) 96:754–66. doi: 10.1212/WNL.0000000000011795 33653902

[7] Rossi S Farina A Malvaso A Dinoto A Fionda L Cornacchini S. Clinical course of neurologic adverse events associated with immune checkpoint inhibitors. Neurol Neuroimmunol Neuroinflammation. (2024) 11(6):e200314. doi: 10.1212/NXI.0000000000200314 PMC11413993 39298719

[8] Berner F Bomze D Diem S Ali OH Fässler M Ring S. Association of checkpoint inhibitor–induced toxic effects with shared cancer and tissue antigens in non–small cell lung cancer. JAMA Oncol. (2019) 5:1043. doi: 10.1001/jamaoncol.2019.0402 31021392 PMC6487908

[9] Bomze D Hasan Ali O Bate A Flatz L. Association between immune-related adverse events during anti–PD-1 therapy and tumor mutational burden. JAMA Oncol. (2019) 5:1633. doi: 10.1001/jamaoncol.2019.3221 31436791 PMC6707013

[10] Khan S Khan SA Luo X Fattah FJ Saltarski J Gloria-McCutchen Y. Immune dysregulation in cancer patients developing immune-related adverse events. Br J Cancer. (2019) 120:63–8. doi: 10.1038/s41416-018-0155-1 PMC6325132 30377338

[11] Dora D Bokhari SMZ Aloss K Takacs P Desnoix JZ Szklenárik G. Implication of the gut microbiome and microbial-derived metabolites in immune-related adverse events: emergence of novel biomarkers for cancer immunotherapy. Int J Mol Sci. (2023) 24(3):2769. doi: 10.3390/ijms24032769 36769093 PMC9916922

[12] Dentoni M Florean I Farina A Joubert B Do LD Honnorat J. Immune checkpoint inhibitor-related cerebellar toxicity: clinical features and comparison with paraneoplastic cerebellar ataxia. Cerebellum. (2024) 23(6):2308–23. doi: 10.1007/s12311-024-01727-5 PMC11585521 39153058

[13] Farina A Birzu C Elsensohn MH Picca A Muñiz-Castrillo S Vogrig A. Neurological outcomes in immune checkpoint inhibitor-related neurotoxicity. Brain Commun. (2023) 5(3):fcad169. doi: 10.1093/braincomms/fcad169 37389303 PMC10306160

[14] Johnson DB Manouchehri A Haugh AM Quach HT Balko JM Lebrun-Vignes B. Neurologic toxicity associated with immune checkpoint inhibitors: a pharmacovigilance study. J Immunother Cancer. (2019) 7:134. doi: 10.1186/s40425-019-0617-x 31118078 PMC6530194

[15] Vogrig A Muñiz-Castrillo S Joubert B Picard G Rogemond V Marchal C. Central nervous system complications associated with immune checkpoint inhibitors. J Neurol Neurosurg Psychiatry. (2020) 91:772–8. doi: 10.1136/jnnp-2020-323055 32312871

[16] Das R Bar N Ferreira M Newman AM Zhang L Bailur JK. Early B cell changes predict autoimmunity following combination immune checkpoint blockade. J Clin Invest. (2018) 128:715–20. doi: 10.1172/JCI96798 PMC5785243 29309048

[17] Che Y Lee J Abou-Taleb F Rieger KE Satpathy AT Chang ALS. Induced B-cell receptor diversity predicts PD-1 blockade immunotherapy response. bioRxiv. (2024) 2024.12.03.626669. doi: 10.1101/2024.12.03.626669 PMC12067265 40314973

[18] Yost KE Satpathy AT Wells DK Qi Y Wang C Kageyama R. Clonal replacement of tumor-specific T cells following PD-1 blockade. Nat Med. (2019) 25:1251–9. doi: 10.1038/s41591-019-0522-3 PMC6689255 31359002

[19] Lozano AX Chaudhuri AA Nene A Bacchiocchi A Earland N Vesely MD. T cell characteristics associated with toxicity to immune checkpoint blockade in patients with melanoma. Nat Med. (2022) 28:353–62. doi: 10.1038/s41591-021-01623-z PMC8866214 35027754

[20] Kuehn HS Ouyang W Lo B Deenick EK Niemela JE Avery DT. Immune dysregulation in human subjects with heterozygous germline mutations in CTLA4. Sci (80-). (2014) 345:1623–7. doi: 10.1126/science.1255904 PMC4371526 25213377

[21] Coustal C Goulabchand R Labauge P Guilpain P Carra-Dallière C Januel E. Clinical, radiologic, and immunologic features of patients with CTLA4 deficiency with neurologic involvement. Neurology. (2023) 101(15):e1560–66. doi: 10.1212/WNL.0000000000207609 PMC10585684 37487754

[22] Havenar-Daughton C Lindqvist M Heit A Wu JE Reiss SM Kendric K. CXCL13 is a plasma biomarker of germinal center activity. Proc Natl Acad Sci. (2016) 113:2702–7. doi: 10.1073/pnas.1520112113 PMC4790995 26908875

[23] Reincke ME Payne KJ Harder I Strohmeier V Voll RE Warnatz K. The antigen presenting potential of CD21low B cells. Front Immunol. (2020) 11:535784. doi: 10.3389/fimmu.2020.535784 33193306 PMC7609862

[24] Farina A Villagrán-García M Fourier A Pinto AL Chorfa F Timestit N. Diagnostic and prognostic biomarkers in immune checkpoint inhibitor-related encephalitis: a retrospective cohort study. Lancet Reg Heal - Eur. (2024) 44:101011. doi: 10.1016/j.lanepe.2024.101011 PMC11338149 39170102

[25] Farina A Villagrán-García M Ciano-Petersen NL Vogrig A Muñiz-Castrillo S Taillandier L. Anti-hu antibodies in patients with neurologic side effects of immune checkpoint inhibitors. Neurol Neuroimmunol Neuroinflammation. (2022) 10(1):e200058. doi: 10.1212/NXI.0000000000200058 PMC9709718 36446613

[26] Déchelotte B Muñiz-Castrillo S Joubert B Vogrig A Picard G Rogemond V. Diagnostic yield of commercial immunodots to diagnose paraneoplastic neurologic syndromes. Neurol Neuroimmunol Neuroinflammation. (2020) 7(3):e701. doi: 10.1212/NXI.0000000000000701 PMC7136063 32170044

[27] Vogrig A Fouret M Joubert B Picard G Rogemond V Pinto AL. Increased frequency of anti-Ma2 encephalitis associated with immune checkpoint inhibitors. Neurol Neuroimmunol Neuroinflammation. (2019) 6(6):e604. doi: 10.1212/NXI.0000000000000604 PMC6705619 31454760

[28] Farina A Villagrán-García M Vogrig A Zekeridou A Muñiz-Castrillo S Velasco R. Neurological adverse events of immune checkpoint inhibitors and the development of paraneoplastic neurological syndromes. Lancet Neurol. (2024) 23:81–94. doi: 10.1016/S1474-4422(23)00369-1 38101905

[29] Chang H Shin Y Keam B Kim M Im S Lee S. HLA-B27 association of autoimmune encephalitis induced by PD-L1 inhibitor. Ann Clin Transl Neurol. (2020) 7:2243–50. doi: 10.1002/acn3.51213 PMC7664281 33031633

[30] Masi G Pham MC Karatz T Oh S Payne AS Nowak RJ. Clinicoserological insights into patients with immune checkpoint inhibitor-induced myasthenia gravis. Ann Clin Transl Neurol. (2023) 10:825–31. doi: 10.1002/acn3.51761 PMC10187728 36924454

[31] O’Hare M Guidon AC. Peripheral nervous system immune-related adverse events due to checkpoint inhibition. Nat Rev Neurol. (2024) 20:509–25. doi: 10.1038/s41582-024-01001-6 39122934

[32] Seki M Kitano S Suzuki S. Neurological disorders associated with immune checkpoint inhibitors: an association with autoantibodies. Cancer Immunol Immunother. (2022) 71:769–75. doi: 10.1007/s00262-021-03053-9 PMC10992020 34515815

[33] Mammen AL Rajan A Pak K Lehky T Casciola-Rosen L Donahue RN. Pre-existing antiacetylcholine receptor autoantibodies and B cell lymphopaenia are associated with the development of myositis in patients with thymoma treated with avelumab, an immune checkpoint inhibitor targeting programmed death-ligand 1. Ann Rheum Dis. (2019) 78:150–2. doi: 10.1136/annrheumdis-2018-213777 PMC6447048 30185415

[34] Suzuki S Ishikawa N Konoeda F Seki N Fukushima S Takahashi K. Nivolumab-related myasthenia gravis with myositis and myocarditis in Japan. Neurology. (2017) 89:1127–34. doi: 10.1212/WNL.0000000000004359 28821685

[35] Müller-Jensen L Knauss S Ginesta Roque L Schinke C Maierhof SK Bartels F. Autoantibody profiles in patients with immune checkpoint inhibitor-induced neurological immune related adverse events. Front Immunol. (2023) 14:1108116. doi: 10.3389/fimmu.2023.1108116 36845122 PMC9945255

[36] Ibis B Aliazis K Cao C Yenyuwadee S Boussiotis VA. Immune-related adverse effects of checkpoint immunotherapy and implications for the treatment of patients with cancer and autoimmune diseases. Front Immunol. (2023) 14:1197364. doi: 10.3389/fimmu.2023.1197364 37342323 PMC10277501

[37] Genta S Lajkosz K Yee NR Spiliopoulou P Heirali A Hansen AR. Autoimmune paneLs as prEdictors of toxicity in patients TReated with immune checkpoint inhibiTors (ALERT). J Exp Clin Cancer Res. (2023) 42:276. doi: 10.1186/s13046-023-02851-6 37865776 PMC10589949

[38] Ghosh N Postow M Zhu C Jannat-Khah D Li QZ Vitone G. Lower baseline autoantibody levels are associated with immune-related adverse events from immune checkpoint inhibition. J Immunother Cancer. (2022) 10:e004008. doi: 10.1136/jitc-2021-004008 35091456 PMC8804686

[39] Abdelhak A Foschi M Abu-Rumeileh S Yue JK D'Anna L Huss A. Blood GFAP as an emerging biomarker in brain and spinal cord disorders. Nat Rev Neurol. (2022) 18:158–72. doi: 10.1038/s41582-021-00616-3 35115728

[40] Barro C Chitnis T Weiner HL. Blood neurofilament light: a critical review of its application to neurologic disease. Ann Clin Transl Neurol. (2020) 7:2508–23. doi: 10.1002/acn3.51234 PMC7732243 33146954

[41] Bjursten S Zhao Z Al Remawi H Studahl M Pandita A Simrén J. Concentrations of S100B and neurofilament light chain in blood as biomarkers for checkpoint inhibitor–induced CNS inflammation. eBioMedicine. (2024) 100:104955. doi: 10.1016/j.ebiom.2023.104955 38171113 PMC10796943

[42] Piepgras J Müller A Steffen F Lotz J Loquai C Zipp F. Neurofilament light chain levels reflect outcome in a patient with glutamic acid decarboxylase 65 antibody–positive autoimmune encephalitis under immune checkpoint inhibitor therapy. Eur J Neurol. (2021) 28:1086–9. doi: 10.1111/ene.14692 33556229

[43] Fonseca E Cabrera-Maqueda JM Ruiz-García R Naranjo L Diaz-Pedroche C Velasco R. Neurological adverse events related to immune-checkpoint inhibitors in Spain: a retrospective cohort study. Lancet Neurol. (2023) 22:1150–9. doi: 10.1016/S1474-4422(23)00335-6 37977714

[44] Vogrig A Muñiz-Castrillo S Farina A Honnorat J Joubert B. How to diagnose and manage neurological toxicities of immune checkpoint inhibitors: an update. J Neurol. (2022) 269:1701–14. doi: 10.1007/s00415-021-10870-6 34708250

[45] Farina A Villagrán-García M Vogrig A Joubert B. Central nervous system adverse events of immune checkpoint inhibitors. Curr Opin Neurol. (2024) 37:345–52. doi: 10.1097/WCO.0000000000001259 38483130

[46] Liu C Chu D Kalantar-Zadeh K George J Young HA Liu G. Cytokines: from clinical significance to quantification. Adv Sci. (2021) 8(15):e2004433. doi: 10.1002/advs.202004433 PMC8336501 34114369

[47] Kartikasari AER Huertas CS Mitchell A Plebanski M. Tumor-induced inflammatory cytokines and the emerging diagnostic devices for cancer detection and prognosis. Front Oncol. (2021) 11:692142. doi: 10.3389/fonc.2021.692142 34307156 PMC8294036

[48] Mao XC Yang CC Yang YF Yan LJ Ding ZN Liu H. Peripheral cytokine levels as novel predictors of survival in cancer patients treated with immune checkpoint inhibitors: A systematic review and meta-analysis. Front Immunol. (2022) 13:884592. doi: 10.3389/fimmu.2022.884592 36072577 PMC9441870

[49] Wang J Ma Y Lin H Wang J Cao B. Predictive biomarkers for immune-related adverse events in cancer patients treated with immune-checkpoint inhibitors. BMC Immunol. (2024) 25:8. doi: 10.1186/s12865-024-00599-y 38267897 PMC10809515

[50] Kauffmann-Guerrero D Kahnert K Kiefl R Sellmer L Walter J Behr J. Systemic inflammation and pro-inflammatory cytokine profile predict response to checkpoint inhibitor treatment in NSCLC: a prospective study. Sci Rep. (2021) 11:10919. doi: 10.1038/s41598-021-90397-y 34035415 PMC8149421

[51] Lim SY Lee JH Gide TN Menzies AM Guminski A Carlino MS. Circulating cytokines predict immune-related toxicity in melanoma patients receiving anti-PD-1–based immunotherapy. Clin Cancer Res. (2019) 25:1557–63. doi: 10.1158/1078-0432.CCR-18-2795 30409824

[52] Mazzarella L Giugliano S D’Amico P Belli C Duso BA Rescigno M. Evidence for interleukin 17 involvement in severe immune-related neuroendocrine toxicity. Eur J Cancer. (2020) 141:218–24. doi: 10.1016/j.ejca.2020.10.006 33186857

[53] Müller-Jensen L Schulz AR Mei HE Mohr R Ulrich C Knape P. Immune signatures of checkpoint inhibitor-induced autoimmunity—A focus on neurotoxicity. Neuro Oncol. (2024) 26:279–94. doi: 10.1093/neuonc/noad198 PMC10836772 37823709

[54] Otto F Seiberl M Bieler L Moser T Kleindienst W Wallner-Essl W. Beyond T cell toxicity – Intrathecal chemokine CXCL13 indicating B cell involvement in immune-related adverse events following checkpoint inhibition: A two-case series and literature review. Eur J Neurol. (2024) 31(7):e16279. doi: 10.1111/ene.16279 38556899 PMC11235827

[55] Li X Zhang S Guo G Han J Yu J. Gut microbiome in modulating immune checkpoint inhibitors. eBioMedicine. (2022) 82:104163. doi: 10.1016/j.ebiom.2022.104163 35841869 PMC9297075

[56] Davar D Zarour HM. Facts and hopes for gut microbiota interventions in cancer immunotherapy. Clin Cancer Res. (2022) 28:4370–84. doi: 10.1158/1078-0432.CCR-21-1129 PMC9561605 35748749

[57] Chaput N Lepage P Coutzac C Soularue E Le Roux K Monot C. Baseline gut microbiota predicts clinical response and colitis in metastatic melanoma patients treated with ipilimumab. Ann Oncol. (2017) 28:1368–79. doi: 10.1093/annonc/mdx108 28368458

[58] Dubin K Callahan MK Ren B Khanin R Viale A Ling L. Intestinal microbiome analyses identify melanoma patients at risk for checkpoint-blockade-induced colitis. Nat Commun. (2016) 7:1–8. doi: 10.1038/ncomms10391 PMC4740747 26837003

[59] Liu T Xiong Q Li L Hu Y. Intestinal microbiota predicts lung cancer patients at risk of immune-related diarrhea. Immunotherapy. (2019) 11:385–96. doi: 10.2217/imt-2018-0144 30693820

[60] Sakai K Sakurai T De Velasco MA Nagai T Chikugo T Ueshima K. Intestinal microbiota and gene expression reveal similarity and dissimilarity between immune-mediated colitis and ulcerative colitis. Front Oncol. (2021) 11:763468. doi: 10.3389/fonc.2021.763468 34778085 PMC8578892

[61] Usyk M Pandey A Hayes RB Moran U Pavlick A Osman I. Bacteroides vulgatus and Bacteroides dorei predict immune-related adverse events in immune checkpoint blockade treatment of metastatic melanoma. Genome Med. (2021) 13:1–11. doi: 10.1186/s13073-021-00974-z 34641962 PMC8513370

[62] Chau J Yadav M Liu B Furqan M Dai Q Shahi S. Prospective correlation between the patient microbiome with response to and development of immune-mediated adverse effects to immunotherapy in lung cancer. BMC Cancer. (2021) 21:1–14. doi: 10.1186/s12885-021-08530-z 34256732 PMC8278634

[63] Cascone T William WN Weissferdt A Leung CH Lin HY Pataer A. Neoadjuvant nivolumab or nivolumab plus ipilimumab in operable non-small cell lung cancer: the phase 2 randomized NEOSTAR trial. Nat Med. (2021) 27:504–14. doi: 10.1038/s41591-020-01224-2 PMC8818318 33603241

[64] Andrews MC Duong CPM Gopalakrishnan V Iebba V Chen WS Derosa L. Gut microbiota signatures are associated with toxicity to combined CTLA-4 and PD-1 blockade. Nat Med. (2021) 27:1432–41. doi: 10.1038/s41591-021-01406-6 PMC11107795 34239137

[65] Hakozaki T Richard C Elkrief A Hosomi Y Benlaïfaoui M Mimpen I. The Gut microbiome associates with immune checkpoint inhibition outcomes in patients with advanced non-small cell lung cancer. Cancer Immunol Res. (2020) 8:1243–50. doi: 10.1158/2326-6066.CIR-20-0196 32847937

[66] McCulloch JA Davar D Rodrigues RR Badger JH Fang JR Cole AM. Intestinal microbiota signatures of clinical response and immune-related adverse events in melanoma patients treated with anti-PD-1. Nat Med. (2022) 28:545–56. doi: 10.1038/s41591-022-01698-2 PMC10246505 35228752

[67] Verheijden RJ van de Wijgert JJHH May AM Viveen M Rogers M Paganelli FL. 771P Correlation of gut microbiome composition with checkpoint inhibitor induced severe immune-related adverse events. Ann Oncol. (2022) 33:S894. doi: 10.1016/j.annonc.2022.07.897

[68] Liu W Ma F Sun B Liu Y Tang H Luo J. Intestinal microbiome associated with immune-related adverse events for patients treated with anti-PD-1 inhibitors, a real-world study. Front Immunol. (2021) 12:756872. doi: 10.3389/fimmu.2021.756872 34975845 PMC8716485

[69] Matson V Fessler J Bao R Chongsuwat T Zha Y Alegre ML. The commensal microbiome is associated with anti–PD-1 efficacy in metastatic melanoma patients. Sci (80-). (2018) 359:104–8. doi: 10.1126/science.aao3290 PMC6707353 29302014

[70] Peters BA Wilson M Moran U Pavlick A Izsak A Wechter T. Relating the gut metagenome and metatranscriptome to immunotherapy responses in melanoma patients. Genome Med. (2019) 11:1–14. doi: 10.1186/s13073-019-0672-4 31597568 PMC6785875

[71] Zheng Y Wang T Tu X Huang Y Zhang H Tan D. Gut microbiome affects the response to anti-PD-1 immunotherapy in patients with hepatocellular carcinoma. J Immunother Cancer. (2019) 7:1–7. doi: 10.1186/s40425-019-0650-9 31337439 PMC6651993

[72] Li L Ye J Liu M. Characterization of gut microbiota in patients with primary hepatocellular carcinoma received immune checkpoint inhibitors: A Chinese population-based study. Med (United States). (2020) 99:E21788. doi: 10.1097/MD.0000000000021788 PMC7489660 32925716

[73] Vétizou M Pitt JM Daillère R Lepage P Waldschmitt N Flament C. Anticancer immunotherapy by CTLA-4 blockade relies on the gut microbiota. Sci (80-). (2015) 350:1079–84. doi: 10.1126/science.aad1329 PMC4721659 26541610

[74] Gopalakrishnan V Spencer CN Nezi L Reuben A Andrews MC Karpinets TV. Gut microbiome modulates response to anti–PD-1 immunotherapy in melanoma patients. Sci (80-). (2018) 359:97–103. doi: 10.1126/science.aan4236 PMC5827966 29097493

[75] Routy B Le Chatelier E Derosa L Duong CPM Alou MT Daillère R. Gut microbiome influences efficacy of PD-1-based immunotherapy against epithelial tumors. Sci (80-). (2018) 359:91–7. doi: 10.1126/science.aan3706 29097494

[76] Huang C Li M Liu B Zhu H Dai Q Fan X. Relating gut microbiome and its modulating factors to immunotherapy in solid tumors: A systematic review. Front Oncol. (2021) 11:642110. doi: 10.3389/fonc.2021.642110 33816289 PMC8012896

[77] Jin Y Dong H Xia L Yang Y Zhu Y Shen Y. The diversity of gut microbiome is associated with favorable responses to anti–programmed death 1 immunotherapy in chinese patients with NSCLC. J Thorac Oncol. (2019) 14:1378–89. doi: 10.1016/j.jtho.2019.04.007 31026576

[78] Davar D Dzutsev AK McCulloch JA Rodrigues RR Chauvin JM Morrison RM. Fecal microbiota transplant overcomes resistance to anti–PD-1 therapy in melanoma patients. Sci (80-). (2021) 371:595–602. doi: 10.1126/science.abf3363 PMC8097968 33542131

[79] Baruch EN Youngster I Ben-Betzalel G Ortenberg R Lahat A Katz L. Fecal microbiota transplant promotes response in immunotherapy-refractory melanoma patients. Sci (80-). (2021) 371:602–9. doi: 10.1126/science.abb5920 33303685

[80] Haanen J Obeid M Spain L Carbonnel F Wang Y Robert C. Management of toxicities from immunotherapy: ESMO Clinical Practice Guideline for diagnosis, treatment and follow-up. Ann Oncol. (2022) 33:1217–38. doi: 10.1016/j.annonc.2022.10.001 36270461

[81] Picca A Valyraki N Birzu C Kramkimel N Hermine O Zahr N. Anti–interleukin-6 and janus kinase inhibitors for severe neurologic toxicity of checkpoint inhibitors. Neurol Neuroimmunol Neuroinflammation. (2021) 8(6):e1073. doi: 10.1212/NXI.0000000000001073 PMC8439960 34497101

[82] Pinal-Fernandez I Quintana A Milisenda JC Casal-Dominguez M Muñoz-Braceras S Derfoul A. Transcriptomic profiling reveals distinct subsets of immune checkpoint inhibitor induced myositis. Ann Rheum Dis. (2023) 82:829–36. doi: 10.1136/ard-2022-223792 PMC10545139 36801811

[83] Dimitriou F Hogan S Menzies AM Dummer R Long GV. Interleukin-6 blockade for prophylaxis and management of immune-related adverse events in cancer immunotherapy. Eur J Cancer. (2021) 157:214–24. doi: 10.1016/j.ejca.2021.08.031 34536945

[84] Manson G Maria ATJ Poizeau F Danlos FX Kostine M Brosseau S. Worsening and newly diagnosed paraneoplastic syndromes following anti-PD-1 or anti-PD-L1 immunotherapies, a descriptive study. J Immunother Cancer. (2019) 7:337. doi: 10.1186/s40425-019-0821-8 31796119 PMC6892018

[85] Petit P-F Daoudlarian D Latifyan S Bouchaab H Mederos N Doms J. Tocilizumab provides dual benefits in treating immune checkpoint inhibitor-associated arthritis and preventing relapse during ICI rechallenge: the TAPIR study. Ann Oncol. (2024) 36(1):43–53. doi: 10.1016/j.annonc.2024.08.2340 39241964

[86] Kang JH Bluestone JA Young A. Predicting and preventing immune checkpoint inhibitor toxicity: targeting cytokines. Trends Immunol. (2021) 42:293–311. doi: 10.1016/j.it.2021.02.006 33714688

[87] Johnson DH Zobniw CM Trinh VA Ma J Bassett RL Jr Abdel-Wahab N. Infliximab associated with faster symptom resolution compared with corticosteroids alone for the management of immune-related enterocolitis. J Immunother Cancer. (2018) 6:103. doi: 10.1186/s40425-018-0412-0 30305177 PMC6180568

[88] Liu J Blake SJ Harjunpää H Fairfax KA Yong MC Allen S. Assessing immune-related adverse events of efficacious combination immunotherapies in preclinical models of cancer. Cancer Res. (2016) 76:5288–301. doi: 10.1158/0008-5472.CAN-16-0194 27503925

[89] Lesage C Longvert C Prey S Maanaoui S Dréno B Machet L. Incidence and clinical impact of anti-TNFα Treatment of severe immune checkpoint inhibitor-induced colitis in advanced melanoma: the mecolit survey. J Immunother. (2019) 42:175–9. doi: 10.1097/CJI.0000000000000268 31090656

[90] Graus F Vogrig A Muñiz-Castrillo S Antoine JG Desestret V Dubey D. Updated diagnostic criteria for paraneoplastic neurologic syndromes. Neurol Neuroimmunol NeuroInflammation. (2021) 8(4):e1014. doi: 10.1212/NXI.0000000000001014 PMC8237398 34006622

[91] Rossi S Gelsomino F Rinaldi R Muccioli L Comito F Di Federico A. Peripheral nervous system adverse events associated with immune checkpoint inhibitors. J Neurol. (2023) 270:2975–86. doi: 10.1007/s00415-023-11625-1 PMC10188572 36800019

[92] Vogrig A Pegat A Villagrán-García M Wucher V Attignon V Sohier E. Different genetic signatures of small-cell lung cancer characterize anti-GABABR and anti-hu paraneoplastic neurological syndromes. Ann Neurol. (2023) 94:1102–15. doi: 10.1002/ana.26784 37638563

[93] Di Liberto G Pantelyushin S Kreutzfeldt M Page N Musardo S Coras R. Neurons under T cell attack coordinate phagocyte-mediated synaptic stripping. Cell. (2018) 175:458–471.e19. doi: 10.1016/j.cell.2018.07.049 30173917

[94] Zak J Pratumchai I Marro BS Marquardt KL Zavareh RB Lairson LL. JAK inhibition enhances checkpoint blockade immunotherapy in patients with Hodgkin lymphoma. Sci (80-). (2024) 384(6702):eade8520. doi: 10.1126/science.ade8520 PMC11283877 38900864

[95] Mathew D Marmarelis ME Foley C Bauml JM Ye D Ghinnagow R. Combined JAK inhibition and PD-1 immunotherapy for non-small cell lung cancer patients. Science. (2024) 384:eadf1329. doi: 10.1126/science.adf1329 38900877 PMC11327955

[96] Ytterberg SR Bhatt DL Mikuls TR Koch GG Fleischmann R Rivas JL. Cardiovascular and cancer risk with tofacitinib in rheumatoid arthritis. N Engl J Med. (2022) 386:316–26. doi: 10.1056/NEJMoa2109927 35081280

[97] Sechi E Markovic SN McKeon A Dubey D Liewluck T Lennon VA. Neurologic autoimmunity and immune checkpoint inhibitors. Neurology. (2020) 95(17):e2442–52. doi: 10.1212/WNL.0000000000010632 PMC7682911 32796130

[98] Yang Y An Y Dong Y Chu Q Wei J Wang B. Fecal microbiota transplantation: no longer cinderella in tumour immunotherapy. eBioMedicine. (2024) 100:104967. doi: 10.1016/j.ebiom.2024.104967 38241975 PMC10831174

[99] Wang Y Wiesnoski DH Helmink BA Gopalakrishnan V Choi K DuPont HL. Fecal microbiota transplantation for refractory immune checkpoint inhibitor-associated colitis. Nat Med. (2018) 24:1804–8. doi: 10.1038/s41591-018-0238-9 PMC6322556 30420754

[100] Chen M Liu M Li C Peng S Li Y Xu X. Fecal microbiota transplantation effectively cures a patient with severe bleeding immune checkpoint inhibitor-associated colitis and a short review. Front Oncol. (2022) 12:913217. doi: 10.3389/fonc.2022.913217 35756645 PMC9229182

[101] Duscha A Gisevius B Hirschberg S Yissachar N Stangl GI Dawin E. Propionic acid shapes the multiple sclerosis disease course by an immunomodulatory mechanism. Cell. (2020) 180:1067–1080.e16. doi: 10.1016/j.cell.2020.02.035 32160527

[102] Chen J Chia N Kalari KR Yao JZ Novotna M Paz Soldan MM. Multiple sclerosis patients have a distinct gut microbiota compared to healthy controls. Sci Rep. (2016) 6:28484. doi: 10.1038/srep28484 27346372 PMC4921909

[103] Tan X Huang Y Chai T Zhao X Li Y Wu J. Differential gut microbiota and fecal metabolites related with the clinical subtypes of myasthenia gravis. Front Microbiol. (2020) 11:564579. doi: 10.3389/fmicb.2020.564579 33013794 PMC7506099

[104] Li K Wei S Hu L Yin X Mai Y Jiang C. Protection of fecal microbiota transplantation in a mouse model of multiple sclerosis. Mediators Inflamm. (2020) 2020:1–13. doi: 10.1155/2020/2058272 PMC7426773 32831634

